# Adjunct therapy with probiotics for chronic urticaria in children: randomised placebo-controlled trial

**DOI:** 10.1186/s13223-021-00544-3

**Published:** 2021-04-17

**Authors:** Xiao-Dong Bi, Bao-Zhen Lu, Xin-Xin Pan, Sha Liu, Jiu-Yao Wang

**Affiliations:** 1 Department of Dermatology, Nanyang First People’s Hospital, Nanyang, 411300 Henan China; 2 Centre for Allergy and Clinical Immunology Research (ACIR), College of Medicine, National Cheng Kung University, Tainan, Taiwan; 3 Children’s Hospital, China Medical University, Taichung, Taiwan; 4 Department of Pediatrics, National Cheng Kung University, No. 138, Sheng-Li Road, Tainan, 70428 Taiwan

**Keywords:** Children, Chronic urticaria, Probiotics, *Lactobacillus*, *Bifidobacillus*

## Abstract

**Backgrounds:**

Chronic urticaria is a common disorder of the skin, characterised by recurrent skin wheals and angioedema. Recent reports have shown that altered diversity and composition of the gut microbiota may lead to imbalances in immune regulation, a causal factor in the occurrence of chronic urticaria.

**Objective:**

This study aimed to evaluate the efficacy of the Yimingjia^®^ probiotic formula in the adjuvant treatment of chronic urticaria in children.

**Methods:**

We enrolled 206 children with confirmed diagnoses of chronic urticaria and randomly assigned them to the treatment (n = 104) or placebo group (n = 102). The children in each group were treated with desloratadine dry suspension, and those in the treatment group also received Yimingjia^®^. Clinical efficacy was evaluated at 1, 2 and 4 weeks.

**Results:**

Clinical symptom scores did not differ significantly at weeks 1 and 2 (p > 0.05), but at 4 weeks, wheal size and attack frequency were significantly reduced in the treatment group (p = 0.049 and 0.03, respectively). The overall response rate (significant improvement + complete response) significantly differed between the treatment (80.8%) and placebo groups (62.5%) (χ^2^ = 4.20, p = 0.04).

**Conclusion:**

Adjunct therapy with Yimingjia^®^ was safe and effective at 4 weeks in the treatment of chronic urticaria in children. The study was registered under trial number NCT03328897.

## Introduction

Urticaria, characterised by wheals (hives), angioedema (in 10%), or both (in 40%), is one of the most common diagnoses in dermatologic practice. Acute urticaria is primarily caused by an allergic or pseudo-allergic reaction to food, medications, or infection. Symptoms often resolve within hours to days and subside fully within 6 weeks. Chronic urticaria, in contrast, presents as daily or episodic wheals of an indeterminate cause that lasts at least 6 weeks [1]. Compared to acute urticaria, chronic disease is more complexly associated with imbalances in immunity, inflammation and coagulation [2]. Despite extensive research, the pathogenesis of chronic urticaria remains largely unknown, though the disease has a dramatic impact on quality of life and treatment poses a major challenge for medical providers. Moreover, paediatric chronic urticaria often occurs with severe pruritus and can affect patients’ physical and mental health [3]. Although non-sedative second generation anti-histamines, though in a higher dose, can effectively relieve symptoms, but these drugs and their long term uses in treating urticaria in children have unwanted side effects.

In the last decade, studies of the human microbiome have shown its close association with immune system development, susceptibility to infectious and chronic diseases, drug response, allergy and even behaviour [4]. Increasing evidence suggests the microbiota that live in and on our bodies are important to human health and disease, although the many functions and consequences of various microbial populations in the body remain poorly understood. Immune system regulation is one of the roles of gut microbiota. Several studies suggest the microbiota mediate allergic diseases such as asthma [5], food allergy [6], and atopic eczema [7]. Changes in the proportions of *Lactobacillus* and *Bifidobacteria* at the beginning of gut microbiome development may have a long-lasting effect on human health and diseases, particularly in allergic diseases such as atopic dermatitis [8–10]. Indeed, there is a significant difference in gut microbiota composition between patients with chronic urticaria and healthy individuals. For example, changes in the composition of *Akkermansia muciniphila*, *Faecalibacterium prausnitzii, Clostridium leptum* and *Enterobacteriaceae* have been reported in patients with chronic urticarial [11]. Pathogenic strains such as *Escherichia coli* are also significantly more prevalent in chronic urticaria, while *Faecalibacterium prausnitzii*, *Prevotella copri* and *Bacteroides *sp. were significantly less prevalent in disease versus healthy controls [12]. These findings of altered gut microbiota in chronic urticaria patients may provide the rationale to correct gut dysbiosis using probiotics supplementation.

Probiotic bacteria, primarily members of the lactic acid bacteria family, are commonly found in decomposing milk products and secrete lactic acid via carbohydrate fermentation. Probiotics are defined as ‘live microorganisms which confer a beneficial effect on the host’ according to the World Health Organization (WHO) [13]. Probiotics may prevent an allergic response due to their anti-inflammatory effects, skewing the Th1/Th2 balance toward Th1 by inhibiting Th2 cytokines or indirectly inducing the production of IL-10 and T-regulatory cells via dendritic cell maturation or toll-like receptors [14]. A recent report showed that a combination probiotic product containing *Lactobacillus* and *Bifidobacteria*, Yimingjia^®^, protected against experimental food allergy via induction of CD103^+^ dendritic cells and modulation of the intestinal microbiota [15]. One of the chief components of Yimingjia^®^, *Lactobacillus gasseri,* has been found to prevent allergen-induced airway inflammation and asthma via PPARα activation in dendritic cells [16] and suppression of the Th17 pro-inflammatory response [17]. This study aimed to evaluate the clinical efficacy and safety of adjunct Yimingjia^®^ therapy in children with chronic urticaria.

## Materials and methods

### Enrolment

During a 12 month period from January to December 2019, we enrolled 206 children aged 6–12 years, with a clinical diagnosis of chronic urticaria and age-matched healthy controls. Chronic urticaria was diagnosed according to the criteria of the American Academy of Allergy, Asthma and Immunology [18]. Briefly, children with chronic urticaria have a disease duration of over 6 weeks, and the onset of symptoms at least twice or 2 days per week, with the duration of each attack within the last 24 h were included. Exclusion criteria were treatment with antibiotics, systemic corticosteroids and other immunosuppressants during one month prior to recruitment, C1 esterase inhibitor deficiency, lymphocytopenia, thrombocytopenia and patients with severe liver, kidney, heart, metabolic diseases and autoimmune diseases. Questionnaires were used to collect demographic information. Criteria for study discontinuation and patient withdrawal included those failed to meet the inclusion/exclusion criteria after review; patients who terminated treatment voluntarily; patients lost to follow-up for any reason; patients who violated the protocol and patients who terminated treatment due to adverse events, although these were included in the evaluation of adverse reactions. The research ethics committee of Nanyang First People’s Hospital, Nanyang, Henan, China, approved the study and written informed were obtained from the parents or guardians of participating children. Procedures and methods involving human subjects were performed in accordance with the Helsinki Declaration in 1964 and its later amendments. The study was registered under trial number NCT03328897.

### Probiotic preparation

The combination probiotic product Yimingia was kindly provided by a pharmaceutical supplier (Ningbo, China) as a lyophilised mixture of six organisms (*Lactobacillus gasseri* LK001, 40%; *Lactobacillus salivarius* LK002, 20%; *Lactobacillus johnsonii* LK003, 15%; *Lactobacillus paracasei* LK004, 5%; *Lactobacillus reuteri* LK005, 5%; *Bifidobacterium animalis* LK011, 15%) at a concentration of 5 × 10^9^ CFU live total bacteria/g. The concentration of each strain is patent-protected. Each strain was isolated from the intestinal tract of normal, healthy neonates at Mother and Child clinics in Tainan, Taiwan, with a high-throughput detection system that exhibits probiotic characteristics. Freeze-dried powder of the mixed probiotics was produced using a high-density culture system (Leu-Kan Biotech. Ningbo, China). The powder was stored at − 4 °C. Each probiotic strain is demonstrated by the National Health Commission of the People’s Republic of China as a strain that can be used for food. Each strain was identified and preserved by the China Centre of Industrial Culture Collection to ensure its safety and purity. The placebo preparation contains the same components as Yimingjia^®^, without the probiotics.

### Study protocol

After diagnosis, patients were randomly and blindly assigned to the treatment or placebo group at a ratio of 1:1 based on a random table. In the treatment group, patients received one satchel (1.5 g) of Yimingjia^®^, twice a day and one bag (2.5 g) of desloratadine dry suspension every night for 4 weeks. Patients in the placebo group also received one bag of desloratadine dry suspension every night and one satchel of a placebo preparation twice daily for 4 weeks. Throughout the treatment period, participants were required to maintain stable doses of the therapy with the addition of H1-antihistamines and/or corticosteroids during exacerbation of urticaria symptoms. The same physician examined each patient at the initial screening visit (W0), and at 1, 2 and 4 weeks (W1–4) after therapy initiation.

### Efficacy evaluation

Throughout the study and one week before starting the probiotic, all patients recorded their symptoms in a daily diary (pruritus and number of wheals). At each visit, the patient was interviewed regarding the previous week/s events and their diary reviewed, followed by a physical examination. The researchers evaluated the patients’ subjective and objective symptoms according to the modified MILOR Study: 1, Pruritus: 0, none; 1: mild, not affecting normal activities; 2: moderate, tolerable, affecting normal activities to some extent; 3: severe, intolerable, significantly affecting activities and sleep. The number of wheals: 0: none; 1: less than 10: 2, 10–25; 3: more than 25. Wheal size (maximum): 0, 0; 1: diameter < 1.5 cm; 2: diameter 1.5–2.5 cm; 3: diameter > 2.5 cm. Duration of each attack: 0: 0 h; 1: < 1 h; 2: 1–12 h; 3: ≥ 12 h. Researchers recorded the number of urticaria attacks per week according to each patient’s diary card or after inquiry. The evaluation of clinical efficacy was based on the total symptom score before and after treatment. The symptom score reduction index (SSRI) was calculated as (symptom score before treatment − symptom score after treatment)/symptom score before treatment. The criteria for evaluating overall efficacy according to SSRI were described as: no improvement: SSRI < 0.20; mild improvement: reduction of 0.20–0.60; significant improvement: reduction of 0.60–0.90; complete response: reduction ≥ 0.90 and the overall response rate to the combination probiotic therapy was the percentage of patients who experienced a complete response or who showed significant improvement.

### Statistical analysis

After completion of all clinical trials, data entry and verification were performed blind and then locked under the supervision of the main researchers, supervisors and statisticians, the statistical personnel opened the sealed random code table to un-blind and group. The principle of intention to treat analysis was utilised. All data were analysed for randomised patients who received at least one dose and had at least one follow-up record. The statistical software SAS6.12 was used, and the two-sided tests, with p ≤ 0.05 having statistical significance.

## Results

**Fig. 1:**
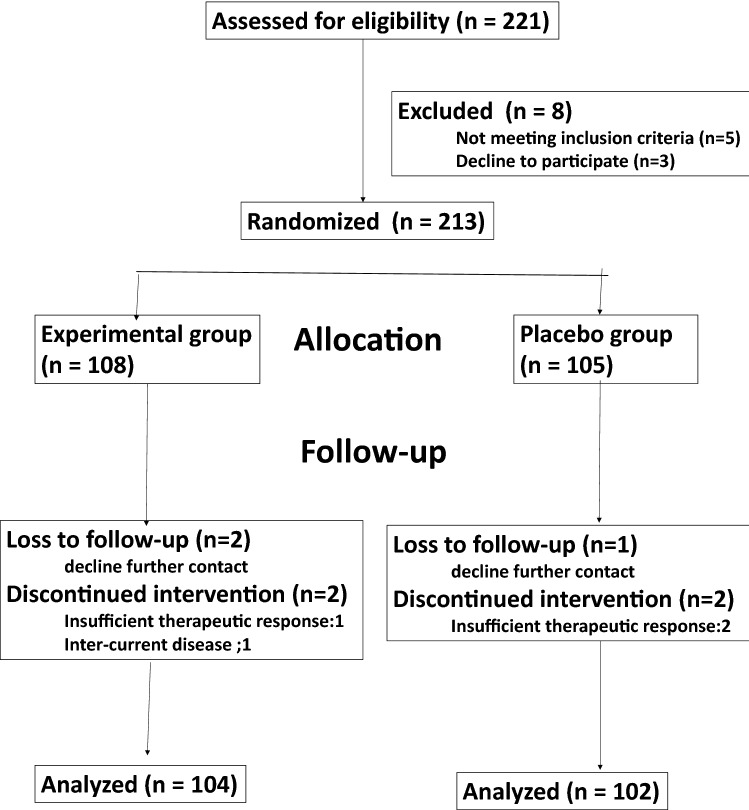
Consort diagram

A total of 213 patients with chronic urticaria were enrolled, 108 of whom were assigned to the treatment group (male 46; female 62; age 6–12 years, with an average age of 8.3 ± 2.4 years; body weight 20.1 ± 12.4 kg; disease duration 1.5–61 months, with an average of 1.8–5.8 months), and the total average symptom score before treatment was 9.1 ± 1.9. Placebo group consists of 105 children (male 50, female 55; age 6–12 years, with an average age of 8.90 ± 2.40 years; body weight 20.10 ± 12.38 kg; disease duration 1.5–67 months with an average of 1.8–5.9 months), and the total symptom score was 8.9 ± 1.8. Statistical analysis showed that the demographic data, disease duration and course severity were comparable between groups (p = 0.09–0.86) (Table 1). Seven patients discontinued treatment or were lost to follow-up, with four cases in the treatment group and three cases in the placebo group, for a discontinuation rate of 5.63%. Ultimately, 206 patients were included in the efficacy statistics of intention to treat analysis, including 104 patients in the treatment group and 102 patients in the placebo group (Fig. 1).

**Table 1:** Baseline demographic characteristics of study participants (n = 213)

Characteristics mean ± sd	Experimental group (n = 108)	Placebo group (n = 105)	p value
Age (years)	8.32 ± 2.41	8.90 ± 2.40	0.75
Gender (m/f)	46/62	50/55	0.86
Body weight (kg)	20.08 ± 12.42	20.10 ± 12.38	0.34
Disease duration (months)	1.82–5.83	1.84–5.87	0.57
Symptom scores before treatment	9.13 ± 1.89	8.94 ± 1.83	0.09

**Fig. 2:**
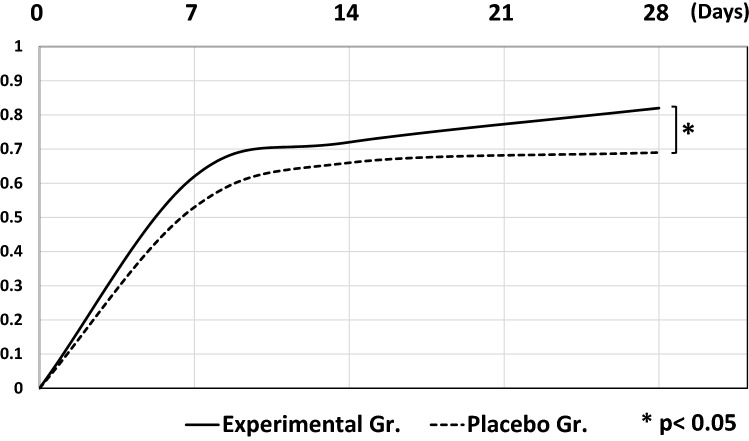
The change of symptom score reduction index (SSRI)

The clinical response evaluation in terms of the SSRI at W1, W2 and W4 (D7, D14, D28) of treatment in the experimental group was 0.62 ± 0.38, 0.72 ± 0.33 and 0.82 ± 0.29, respectively, and 0.53 ± 0.41, 0.66 ± 0.34 and 0.69 ± 0.31 in the placebo group. Thus, both groups experienced relief in subjective and objective symptoms of chronic urticaria over the observation period, while the rank-sum test indicated a significant difference between groups at week 4 (p = 0.04) (Fig. 2). At the end of treatment (W4), the results of a 4-grade overall efficacy analysis, according to SSRI, are shown in Table 2. The difference in the percentage of clinical efficacy (significant improvement and complete response) between the treatment (84/104, 80.8%) and placebo group (63/102, 61.7%) was significant (p < 0.05).

**Table 2:** Efficacy on chronic urticaria in the experiment group and the placebo group

Group	Number of subjects	No improvement	Mild improvement	Significant improvement	Complete response	Overall response rate (%)^a,b^
Experiment group	104	7 (6.7%)	13 (12.5%)	16 (15.4%)	68 (65.4%)	84 (80.8%)
Placebo group	102	7 (6.9%)	32 (31.3%)	21 (20.6%)	42 (41.1%)	63 (61.7%)

^a^Overall response rate = significant improvement + complete response

^b^The overall response rate was evaluated by χ^2^ test

p < 0.05 between these two groups

The clinical observation index scores before and after treatment in both groups are shown in Table 3. There was a significant improvement in the scores for pruritus, the number of wheals and duration of urticaria attacks in each group before and after treatment at W4 (day 28), but no significant differences were found between groups (p = 0.10, 0.5 and 0.68, respectively). In contrast, the scores for wheal size and frequency of attacks per week, although reduced significantly in each group before and after treatment at W4 (day 28), were significantly lower in the treatment versus the placebo group (Z = 1.94, p = 0.049 and Z = 2.15, p = 0.03, respectively).

**Table 3:** The scores of observation indexes before and after treatment of chronic urticaria in the experiment group and the placebo group (means ± sd)

Group	Before treatment	After treatment		
		W1 (day 7)	W2 (day 14)	W4 (day 28)
Pruritus				
Experiment	2.19 ± 0.66	0.80 ± 0.85	0.53 ± 0.72	0.39 ± 0.67
Placebos	2.19 ± 0.68	1.00 ± 0.92	0.66 ± 0.76	0.46 ± 0.77
Number of wheals				
Experiment	2.44 ± 0.63	0.70 ± 0.89	0.42 ± 0.80	0.90 ± 1.01
Placebos	2.40 ± 0.67	0.84 ± 0.90	0.58 ± 0.09	1.09 ± 1.05
Size of wheals				
Experiment	2.27 ± 0.79	1.01 ± 0.96	0.63 ± 0.82	0.39 ± 0.72^#^,*
Placebos	2.39 ± 0.69	1.06 ± 1.01	0.66 ± 0.89	0.52 ± 0.79
Duration of attack				
Experiment	2.05 ± 0.64	0.90 ± 0.99	0.66 ± 0.83	0.42 ± 0.72
Placebos	2.15 ± 0.73	1.03 ± 1.00	0.83 ± 0.87	0.48 ± 0.77
Attacks per week				
Experiment	7.13 ± 3.06	4.31 ± 3.96	3.01 ± 3.57	1.03 ± 3.96^#^,**
Placebos	7.73 ± 5.74	4.91 ± 5.27	3.43 ± 4.26	2.51 ± 4.17

^#^On Day 7 after beginning treatment, the wheal size score and attacks per week between the experiment group and the placebo group separately were compared by the rank-sum test, and the results were Z = 1.94, p = 0.05 (*) and Z = 2.15, p = 0.02 (**), respectively

## Discussion

This randomised placebo-controlled study showed that, in addition to conventional therapeutics with long-acting antihistamines, supplementation with the Yimingjia^®^ combination probiotic improved the SSRI versus placebo in children with chronic urticaria over 4 weeks with no adverse effect. The overall response rate in the treatment group was 80.8%, significantly better than the 62.5% observed in the placebo group (p < 0.05, Table 2). Moreover, we found that probiotic supplementation reduced wheal size and attack frequency from the first week of treatment until the end of the 4-week study (Table 3).

The aetiology of chronic urticaria is not clear, particularly in children. Immune dysregulation, histamine release and mast cell degranulation are suggested underlying mechanisms. In a previous study, Th1 and Th2 cytokine expression closely correlated with disease severity, indicating that a Th1/Th2 imbalance is involved in the pathogenesis of urticaria disease [19]. Recently, increased Th17 and IL-17 expression in both CD4 ^+^ T cells and mast cells was found in the skin of patients with severe chronic urticaria, and increased serum IL-17 was associated with disease severity [20]. These findings suggest that aberrant cytokine expression and dysfunction of regulatory T cells are common in patients with chronic urticarial [21].

Recent studies have demonstrated that immune regulation via the gut microbiome has a role in chronic urticaria. Indeed, the role of the gut microbiome in the immune regulation of allergic diseases has been demonstrated in asthma [5], food allergy [22] and atopic eczema [23]. Recently, Lu et al. [12] demonstrated that gut microbial populations significantly differ between patients with chronic urticaria and healthy individuals. Pathogenic strains including *Escherichia coli* are significantly more prevalent in chronic urticaria, while *Prevotella copri*, *Faecalibacterium prausnitzii* and *Bacteroides *sp. were significantly less prevalent. In a comparison of gut microbiota and serum metabolites in patients with chronic spontaneous urticaria and healthy controls, Wang et al. [24] found patients with chronic spontaneous urticaria had profound changes in the composition of gut microbes–metabolites. The disease group exhibited decreased alpha diversity and the abundance of unidentified *Enterobacteriaceae* was increased, while the abundance of *Bacteroides, Faecalibacterium, Bifidobacterium* and unidentified *Ruminococcaceae* was significantly reduced in patients with chronic spontaneous urticaria. Serum metabolome analysis revealed altered levels of docosahexaenoic acid, arachidonic acid, glutamate and succinic acid, suggesting changes in unsaturated fatty acids and the butanoate metabolism pathway. Although the microbiota composition and diversity reduction on the pathogenesis of chronic urticaria are not well defined, however, the dysbiosis of gut microbiota in chronic urticaria patients provide the rationale for modification of gut microbiota via the provision of probiotics and/or prebiotics [25].

There have been few reports of probiotic supplementation in small numbers of patients with chronic urticarial [11, 12, 26, 27]. Nettis et al. [27] investigated the clinical efficacy and safety of 8 weeks twice daily oral *Lactobacillus salivarius* and *Bifidobacterium breve* in 52 Italian patients (10 male and 42 female, age 19–72 years) with chronic spontaneous urticaria who remained symptomatic despite H1-antihistamine therapy. During the study, 14 patients discontinued therapy and, of the 38 remaining patients, 23.7% experienced mild clinical improvement while 2.6% reported significant improvement and complete remission was reported in 2.6% of patients. However, 71.1% of patients did not experience an improvement in symptoms. The researchers concluded that this therapeutic method might reduce symptoms and improve quality of life for those patients who remained symptomatic despite treatment with H1 antihistamine [27]. Indeed, not all probiotic supplements provide similar beneficial results in allergic disorders [25]. A lack of uniformity in genera, species, strains and doses of probiotics obtained in different settings and/or populations, presumably with variations in their native intestinal microbiota, may result in misleading conclusions.

In this study, a combination probiotic product of six organisms (five *Lactobacillus* strains and one *Bifidobacterium* strain) was administered orally twice daily for 4 weeks in children with chronic urticaria. The safety profile in the treated subjects was consistent with previous observations in children with asthma treated with a similar probiotic product [8]. Yimingjia^®^ also suppressed OVA-induced food allergy in an experimental mouse model and in the maintenance of the tolerogenic function of CD103 ^+^ dendritic cells and microbiome homeostasis, all of which is involved in IgA responses and immune tolerance in the gut [28]. Therefore, we suspect that administration of Yimingjia^®^ helps by maintaining the microbiome homeostasis in the gut of patients with chronic urticaria, and provides immune tolerance to dietary foods as well as induction of regulatory T (Treg) cells [29, 30]. This proposed mechanism is supported by studies that showed a reduced number [31] and function [32] of Treg cells in patients with chronic urticaria. Other suggested benefits of these probiotic organisms in adjunct therapy include improving the composition of the intestinal microbiota by lowering the pH level, modulating the integrity of the epithelial barrier, regulation of the mucus secretion and altering the function and expression of tight junction proteins [33, 34]. It has been postulated that a collection of functionally distinct bacterial species rationally selected from the human gut microbiome may be more effective than single strains in preventing/treating disease [35], supporting our hypothesis that the combination six-product strain used here may be more effective than any of the strains alone. In a future study, we will compare the therapeutic efficacy of the combination product versus each strain independently. Additional in vitro and in vivo studies are needed, as well as a complete analysis of the intestinal microflora to understand the protective effect of blended probiotics in patients with chronic urticaria.

This study was limited in its duration, so the long-term effect of adjuvant therapy for chronic urticaria with probiotics remains unknown. Additional studies are needed to identify clinical biomarkers and laboratory predictors such as microbiome clustering [12] to optimise therapeutic efficacy. Despite these limitations, our data showed that supplementation of standard long-acting antihistamine therapy with Yimingjia^®^ for 4 weeks was effective and safe for children with chronic urticaria. More clinical studies are needed to evaluate whether this approach will provide a new well-tolerated option in the management of patients with chronic urticaria.

## Conclusions

Our study supports that probiotics combination is beneficial as adjunct therapy for children with chronic urticaria. We found that probiotics combination accompanied with long acting anti-histamine medication for four weeks can reduce clinical symptom score, wheal size and attack frequency in school-age children as compared to those with medication alone. This adjunct therapy with probiotics combination were well tolerated with fair compliance and without adverse effects reported.

## Data Availability

All information in this clinical trials and the raw data supporting the conclusions of this article will be made available by the authors, without undue reservation, to any qualified researcher.
